# Development of a Simultaneous Normal-Phase HPLC Analysis of Lignans, Tocopherols, Phytosterols, and Squalene in Sesame Oil Samples

**DOI:** 10.3390/foods13091368

**Published:** 2024-04-28

**Authors:** Jitkunya Yuenyong, Chonlada Bennett, Sudarat Jiamyangyuen, Sugunya Mahatheeranont, Phumon Sookwong

**Affiliations:** 1 Rice and Cereal Chemistry Research Laboratory, Department of Chemistry, Faculty of Science, Chiang Mai University, Chiang Mai 50200, Thailand; jitkunya_y@cmu.ac.th (J.Y.); chonlada.b@cmu.ac.th (C.B.); sugunya.m@cmu.ac.th (S.M.); 2 The Graduate School, Chiang Mai University, Chiang Mai 50200, Thailand; 3 Material Science Research Center, Faculty of Science, Chiang Mai University, Chiang Mai 50200, Thailand; 4 Division of Food Science and Technology, Faculty of Agro-Industry, Chiang Mai University, Chiang Mai 50100, Thailand; sudarat.j@cmu.ac.th; 5 Center of Excellence for Innovation in Chemistry, Faculty of Science, Chiang Mai University, Chiang Mai 50200, Thailand; 6 The Functional Food Research Center for Well-Being, Multidisciplinary Research Institute, Chiang Mai University, Chiang Mai 50200, Thailand

**Keywords:** HPLC-DAD-FLD, simultaneous analysis, *Sesamum indicum*, phytochemicals, method validation, quantitative analysis, method development

## Abstract

The objective of this study was to develop a simultaneous analytical method for the determination of lignans, tocols, phytosterols, and squalene using high-performance liquid chromatography coupled with a diode array and fluorescence detector (HPLC-DAD-FLD). The method employed a Vertisep^TM^ UPS silica HPLC column (4.6 × 250 mm, 5 µm) with a mobile phase mixture of n-hexane/tetrahydrofuran/2-propanol. This approach enabled the simultaneous analysis of ten compounds within 22 min. The linear correlation (R^2^) exceeded 0.9901. The limit of detection (LOD) and limit of quantitation (LOQ) were up to 0.43 µg mL^−1^ for lignans and tocopherols and up to 326.23 µg mL^−1^ for phytosterol and squalene. The precision and accuracy of the intra-day and inter-day variation were less than 1.09 and 3.32% relative standard deviations (RSDs). Furthermore, the developed method was applied for the analysis of targeted compounds in twenty-eight sesame oil samples (1775–8965 µg g^−1^ total lignans, 29.7–687.9 µg g^−1^ total tocopherols, 2640–9500 µg g^−1^ phytosterol, and 245–4030 µg g^−1^ squalene). The HPLC method that has been developed was proven to be a reliable and effective tool for the determination of those functional compounds among sesame oil samples.

## 1. Introduction

*Sesamum indicum* L., commonly known as sesame, is an annual herbaceous plant belonging to the Pedaliaceae family. The seeds come in a small, flat, oval shape, and exhibit a range of colors from white to black, which is contingent upon the particular cultivar. The sesame plant is one of the most important oilseed crops cultivated for essential dietary oils in Asian (such as China, Korea, Japan, Thailand, and India) and Western countries (such as France, Portugal, Spain, and Mexico) [1,2]. In general, cooking sesame oil is derived from solvent extraction with oil refining processes, involving filtering, bleaching, and deodorizing to remove impurities and enhance the stability of the oil. Refined sesame oil has a light color, mild flavor, and higher smoke point, making it suitable for cooking, frying, and salad dressings [3]. Another type of unrefined sesame oil, also known as virgin sesame oil or cold-pressed sesame oil (CPO), is the oil extracted from sesame seeds using mechanical pressing methods without any chemical refining. It retains more of the natural flavor, aroma, and nutrients of the sesame seeds, making it ideal for use in traditional cooking, flavoring, and medicinal applications [4]. Compared with other oils, sesame oil is highly valued in food products and medicinal and cosmetic applications due to its superior quality, rich functional compound content, unique flavor, and fragrance [5,6,7].

Key components in sesame oil are lignans, including sesamin, sesamolin, sesamol, and asarinin. These compounds have been reported to offer various health benefits such as lowering cholesterol levels through the inhibition of absorption and biosynthesis, as well as demonstrating antihypertensive and anticancer properties [8,9]. Sesame oil also contains other functional compounds including tocopherols, phytosterols, and squalene [5,6]. The oxidative stability of sesame oil is attributed to its antioxidant properties, particularly the tocopherol-sparing effects, hypotensive effects, and anti-aging effects. Among the different tocopherol isoforms, γ-tocopherol is the predominant tocopherol in sesame oil, with δ-tocopherol accounting for less than 5% of the total tocopherols and β-tocopherol being present in trace amounts [5]. Sesame oil is one of the richest oil sources of phytosterols containing as much as 1.9% of the total sterols, in which β-sitosterol is the most abundant sterol. Consumption of phytosterol may decrease cholesterol absorption in the intestine and lower serum low-density lipoprotein-cholesterol concentration, leading to anti-inflammatory benefits and the prevention of chronic disorders such as cardiovascular diseases and diabetes [10]. Several studies have indicated that squalene is an important dietary cancer chemopreventive agent [11,12]. The protective effects of squalene may be attributed to its capacity to enhance cellular antioxidant levels [13]. Therefore, to maximize the health advantages, it is necessary to conduct a quantitative and qualitative analysis of the functional compounds present in sesame oil.

HPLC is a chromatographic technique effective for the separation, identification, and quantification of individual components in complex mixtures depending on the type of stationary phase employed, mobile phase (solvent), stationary phase (column), sample injector, detector, and data acquisition system [14]. Until now, HPLC has enabled the separation and quantification of lignan groups from complex matrices such as seeds, grains, and plant extracts [15]. HPLC with ultraviolet (UV) or fluorescence detection allows for the precise determination of tocopherol isoforms (α, β, γ, δ) in various food and biological samples [16]. HPLC with a suitable detector (e.g., UV, evaporative light scattering) enables the separation and quantification of phytosterols in food products, dietary supplements, and biological samples [17]. Although the high capability of HPLC for the determination of each compound group was well documented, the development of a single HPLC system for the simultaneous determination of lignans, tocopherols, phytosterols, and squalene in a single run is still very challenging.

To the best of our knowledge, there has been no simultaneous analysis of lignans, tocopherols, phytosterols, and squalene in sesame oils that can quantify each compound accurately and precisely. This could potentially be achieved by selecting a suitable separation column, choosing an appropriate mobile phase, and combining the detection of suitable detectors for targeted compounds. For instance, Pokkanta et al., developed an HPLC method that utilized a hydrophobic phase column modified with pentafluorophenyl-propyl groups, along with fluorescence and diode array detectors, to simultaneously separate and quantify tocopherols, γ-oryzanols, phytosterols, squalene, cholecalciferol, and phylloquinone in vegetable oil [18]. Accordingly, this study had an objective to develop an HPLC method for the efficient separation and quantification of the targeted compounds in sesame oil samples, including lignans (sesamin, sesamolin, asarinin, sesamol), tocopherols (α-, β-, γ-, and δ-tocopherol), phytosterols, and squalene. After the HPLC method was developed, method validation was performed to certify the usability and effectiveness of the method, including linearity of the calibration curve, accuracy, limit of detection (LOD), limit of quantification (LOQ), and instrument precision. Subsequently, the developed method was applied for the determination of targeted compounds in twenty-eight different sesame oil samples.

## 2. Materials and Methods

### 2.1. Chemicals

Standard sesamin (PubChem CID: 72307, purity ≥ 98%) and sesamolin (PubChem CID: 101746, purity ≥ 98%) were purchased from Biopurity Phytochemicals Ltd. (Chengdu, China). Sesamol (PubChem CID: 68289, purity ≥ 97%) was purchased from Sigma Aldrich Corp., Ltd. (St. Louis, MO, USA). L-Asarinin (PubChem CID: 1869417, purity ≥ 98%) was purchased from Toronto Research Chemicals (TRC, North York, ON, Canada). Standard α-, β-, γ-, and δ-tocopherols (PubChem CID: 14985, 6857447, 92729, and 92094, respectively, purity ≥ 95%) were purchased from Eisai Food & Chemical Co., Ltd. (Tokyo, Japan). Standard phytosterols (PubChem CID: 87575667, purity ≥ 95%) and squalene (PubChem CID: 637072, purity ≥ 98%) were purchased from Tokyo Chemical Industry Co., Ltd. (Tokyo, Japan). HPLC-grade solvents including n-hexane (PubChem CID: 8058), tetrahydrofuran (PubChem CID: 8028), acetonitrile (PubChem CID: 6342), and 2-propanol (PubChem CID: 3776) were purchased from RCI Labscan Co., Ltd. (Bangkok, Thailand).

### 2.2. Sesame Oil Samples

Twenty-eight commercialized sesame oil samples were purchased from the local markets and the internet in Thailand. The oils were categorized into 4 groups, including cold-pressed sesame oil (CPO, 7 samples), sesame oil supplements (SOS, 4 samples), sesame cooking oil (SCO, 12 samples), and sesame cooking oil mixed with other oils (MSCO, 5 samples). The sesame oil products were manufactured in different countries including Thailand (16 samples), China (6 samples), Korea (2 samples), Singapore (2 samples), Japan (1 sample), and India (1 sample). Details of the sample oils (such as type of sesame, pre-treatment of sesame seeds, and composition of other vegetable oils) are described in Supplementary Table S1. The oils were stored below 4 °C in a refrigerator prior to analysis.

### 2.3. Sample Preparation

Sesame oil samples (0.010 g) were weighed into a 10 mL volumetric flask and diluted with n-hexane to make a final volume of 10.00 mL. The solution was filtered through a 0.45 µm syringe nylon filter before HPLC analysis.

### 2.4. HPLC-DAD-FLD Analysis

The HPLC system consisted of an Agilent HPLC 1100 connected to a diode array detector (DAD, model G1315 A, Agilent Technologies, Palo Alto, CA, USA) and a fluorescence detector (FLD, model 1046A, Hewlett Packard, Palo Alto, CA, USA). The separation column used a Vertisep^TM^ UPS silica HPLC column (4.6 × 250 mm, 5 µm, Vertical Chromatography Co., Ltd., Bangkok, Thailand). The mobile phase consisted of n-hexane, tetrahydrofuran, 2-propanol, and acetonitrile. The column temperature was set at 30 °C and the flow rate was 0.8 mL min^−1^. Lignans and tocopherols were detected in FLD with an excitation wavelength of 295 nm and an emission wavelength of 330 nm. DAD was used for the quantification of phytosterols and squalene at 210 nm. The concentrations of the targeted compounds were calculated with the calibration curve of their standards and the quantitative data were represented as µg g^−1^ of the sample. 

### 2.5. Method Validation

The external standard method was used to obtain the standard curves using five to ten concentrations for each compound. The standard curves were validated to ensure the suitability of the curve for quantitative analysis. The parameters to be evaluated included linearity, LOD, LOQ, recovery, and precision (intra-day and inter-day). A correlation coefficient (R^2^) of more than 0.9900 was considered acceptable for accessing the linearity of the curves, indicating a very good fit of the standard curves to the data. The LOD and LOQ are the terminologies used for assessing the method’s ability to detect and quantify the lowest concentrations of analytes with acceptable accuracy and precision. These can be performed by measuring the signal (S) generated by the analyte at low concentrations and comparing it to the background noise (N) level. Signal-to-noise ratios (S/N) of 3 and 10 were set as criteria for LOD and LOQ, respectively. Precision was measured as the repeatability of the method by one operator using the same instrument on the same day for intra-day precision (6 replicates × 1 day) and on 3 separate days for inter-day precision (3 replicates × 3 days). The repeatability of the method was defined by the relative standard deviation (RSD) for each compound. To determine the accuracy (recovery), sesame oil samples (CPO-4) were spiked using a known concentration of standards. The recovery was quantified as a percentage of the spiked amount. The acceptance criteria for recoveries were 85–115%. The range represents a reasonable balance ensuring effective sample retrieval while accommodating possible losses or variation throughout the analytical procedure [19].

### 2.6. Statistical Analysis

Principal component analysis (PCA) is a statistical technique used to summarize and visualize large datasets. PCA modeling was achieved based on the algorithm where the collection of new variables created by PCA from original datasets was used to identify the primary and significant variation in the data [20]. According to a study by Brereton, R. G., the mathematical transformation of an original data matrix (X) using PCA can be described by the following equation.

X = TP + E

According to the equation, T is the score matrix, representing the relationship among the studied samples. P is the loading matrix, visualizing the behavior among the parameters. E is the residual matrix, containing the variation excluded from the analysis. On the PCA score plot, samples with similar score values appeared close to one another, demonstrating that they have a similarity or share a relationship within the dataset. Standardization scaling was applied to preprocess the data during analysis to ensure that the measured intensity of the variables was adjusted to the same scale [21]. All quantitative data were obtained in triplicates (*n* = 3) and expressed as the mean ± standard deviation. Statistical analysis used a one-way ANOVA with Minitab (version 21.4.1, trial version, Minitab LLC, State College, PA, USA). Significant differences were assessed by post-hoc Tukey’s test with the level of significance at 95% (*p* < 0.05).

## 3. Results

According to the literature, both reverse-phase (RP) and normal-phase (NP) HPLC columns have the potency to separate targeted compounds in sesame oils. A previous study using the RP-HPLC C18 column and gradient elution of methanol and water as the mobile phase separated sesamol, sesamin, sesamolin, α-tocopherol, γ-tocopherol, and δ-tocopherol, but not asarinin or β-tocopherol in sesame oil [22]. Another study by Yan et al. utilized the RP-HPLC C18 column, a mixture of methanol/water (90:10, *v*/*v*) as the mobile phase, and 290 nm ultraviolet/visible detection for the separation of functional compounds in sesame oil. The RP-HPLC method required a complicated pretreatment of oil samples such as saponification and extraction and could not completely separate β- and γ-tocopherols [23]. While the RP-HPLC-based method developed by Pokkanta et al. demonstrated the ability to separate various groups of functional compounds such as tocopherols, phytosterols, squalene, cholecalciferol, and phylloquinone [18], it was found to be unsuitable for separating lignan. In many cases, RP-HPLC analysis of lignan or tocopherol compounds needed approximately 30 min for a single run [22,23]. Achieving simultaneous determination of tocopherol and lignan in sesame oil is laborious and cost intensive [24]. On the other hand, NP-HPLC [25,26] requires simple pretreatment and has the capability to completely separate the four tocopherol isoforms (α-, β-, γ-, and δ-tocopherols) and lignans (sesamin, sesamolin, sesamol, and asarinin) in sesame oil. The method largely shortens the detection time and cost. Therefore, on the basis of these findings, this study aims to develop an NP-HPLC method for the separation of a wider spectrum of the bioactive compounds in sesame oils, including tocopherols and lignans with the addition of phytosterol and squalene. The method incorporated a simple pretreatment of the sample and a comparatively short analytical time.

### 3.1. Development of Simultaneous Determination of Lignans, Tocopherols, Phytosterols, and Squalene

**Figure 1:**
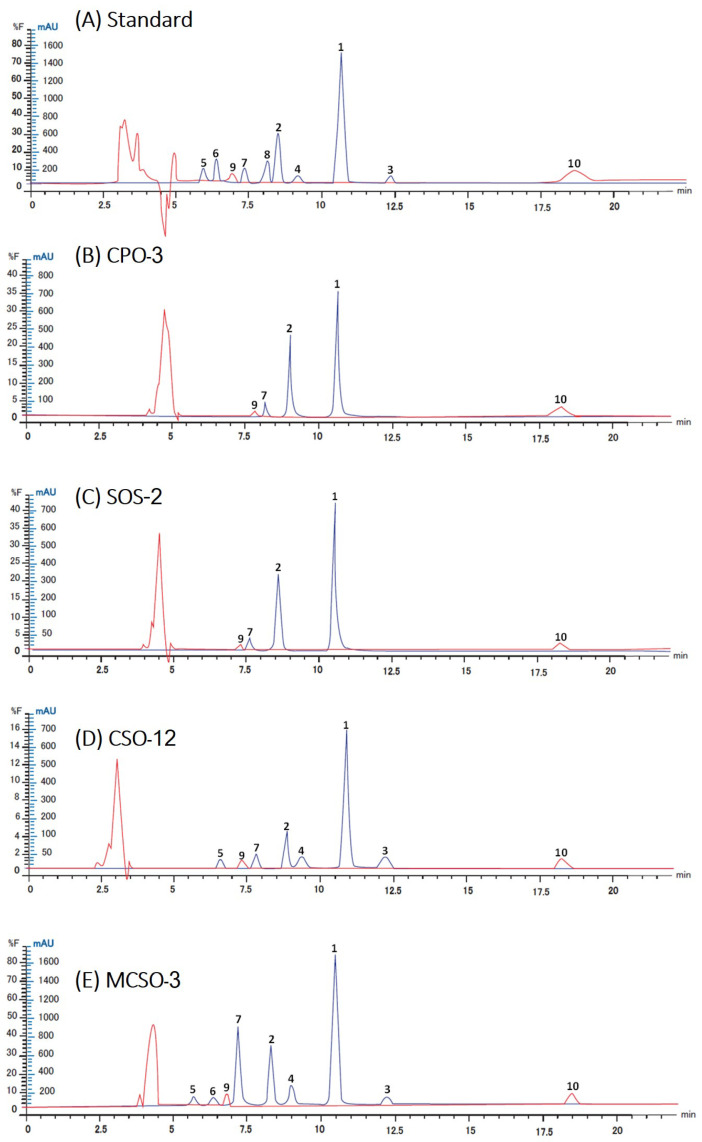
Chromatograms of analyzed samples. (**A**) Standard solution, (**B**) cold-pressed sesame oil (CPO), (**C**) sesame oil supplements (SOS), (**D**) sesame cooking oil (SCO), and (**E**) sesame cooking oil mixed with other oils (MSCO). Peak identification: 1 = sesamin, 2 = sesamolin, 3 = sesamol, 4 = asarinin, 5 = α-tocopherol, 6 = β-tocopherol, 7 = γ-tocopherol, 8 = δ-tocopherol, 9 = squalene, and 10 = phytosterol.

In the present study, NP-HPLC was used for the simultaneous analysis of tocopherols, lignans, phytosterols, and squalene in sesame oil samples, employing a conventional silica column with a dimension of 4.6 × 250 mm (5 µm particle size). The mobile phases tested were various compositions of n-hexane/tetrahydrofuran/acetonitrile/2-propanol mixture with isocratic elution of 0.5 to 1.2 mL min^−1^. Some mobile phase ratios that showed effectiveness in the separation of the targeted compounds were as follows: condition A, a mixture of n-hexane/tetrahydrofuran/acetonitrile/2-propanol (93:6:0.5:0.5, *v*/*v*/*v*/*v*) using a flow rate of 0.8 mL min^−1^; condition B, a mixture of n-hexane/tetrahydrofuran/acetonitrile/2-propanol (92:6:1:1, *v*/*v*/*v*/*v*) using a flow rate of 0.5 mL min^−1^; condition C, a mixture of n-hexane/tetrahydrofuran/acetonitrile (93:6:1, *v*/*v*/*v*) using a flow rate of 1.2 of mL min^−1^, and condition D, a mixture of n-hexane/tetrahydrofuran/2-propanol (93:6:1, *v*/*v*/*v*) using a flow rate of 0.8 of mL min^−1^. Chromatograms derived from the four conditions are shown in Supplementary Figure S1. Considering condition A, four tocopherols were separated but peaks of sesamin and asarinin were co-eluted (Supplementary Figure S1A). In condition B, the separation time was extended to 25 min, with the peaks of sesamolin and δ-tocopherol overlapped, and the peaks of sesamin and asarinin inseparable (Supplementary Figure S1B). Separation with condition C could separate all compounds except for sesamin and asarinin (Supplementary Figure S1C). Consequently, separation with a mobile phase of n-hexane/tetrahydrofuran/2-propanol (condition D) could successfully separate all four tocopherols and four lignans within 15 min (FLD), and squalene as well as phytosterols (DAD) within 20 min (Supplementary Figure S1D). Therefore, condition D (n-hexane/tetrahydrofuran/2-propanol (93:6:1, *v*/*v*/*v*) using a flow rate of 0.8 of mL min^−1^ was chosen as the optimized separation condition for the simultaneous analysis of bioactive compounds in sesame oils. The elution order was α–tocopherol (6.31 min) > β–tocopherol (6.97 min) > squalene (7.38 min) > γ–tocopherol (7.52 min) > δ–tocopherol (8.44 min) > sesamolin (8.94 min) > asarinin (9.08 min) > sesamin (10.34 min) > sesamol (11.73 min) > phytosterol (18.17 min) (Figure 1). The developed method was used for the simultaneous determination and quantification of lignans, tocopherols, phytosterols, and squalene in twenty-eight sesame oil samples (Section 3.3).

### 3.2. Method Validation

The calibration equations, linearity assessment, intra-day and inter-day precisions, LOD, LOQ, and recovery are listed in Table 1 and Table 2.

#### 3.2.1. Linearity, LOD, and LOQ

Linearity refers to the ability of an analytical method to produce results that are directly proportional to the concentration of the analyte within a specified range. The LOD and LOQ are the limits at which the lowest concentration can be reliably detected by the analytical method. In analytical procedures, the LOD and LOQ are based on the signal-to-noise ratio (S/N) of 3 and 10, respectively, in relation to the baseline noise. The LODs in FLD were 0.02–0.41 µg mL^−1^ for lignans and tocopherols, and 10.04–307.07 µg mL^−1^ for phytosterol and squalene in DAD. The LOQs were 0.04–0.43 µg mL^−1^ for lignans and tocopherols and 10.34–326.23 µg mL^−1^ for phytosterol and squalene with a correlation (R^2^) of more than 0.9901 (Table 1).

#### 3.2.2. Precision and Accuracy

The precision and accuracy of the method were the relative standard deviations (RSDs) of the intra-day and inter-day variation of three different concentrations (Table 3). The RSDs of the intra-day and inter-day precision were less than 1.09% and 3.32% for lignans, 0.33% and 0.45% for tocopherols, 0.04% and 0.25% for phytosterol, and 0.22% and 0.46% for squalene, respectively. The results indicated that the precision and accuracy of the whole method were good.

#### 3.2.3. Recovery

The recoveries of lignans, tocopherols, squalene, and phytosterol were measured using sesame oil samples spiked with three standard concentrations of analytes (Table 2). The spiked recoveries were 88.4–107.2% for lignans, 88.9–113.5% for tocopherols, 91.0–94.6% for phytosterol, and 99.7–104.7% for squalene, respectively. The RSDs of the intra-day and inter-day precisions were less than 2.50 and 3.74% for tocopherols, and 2.83 and 3.77% for lignans, respectively [25]. The recoveries of the analytes were 96.0–102.9% considering linearity assessment [18]. This range indicates the accuracy of the measurement method used to determine the presence and concentration of lignans, tocopherols, squalene, and phytosterols in sesame oil spiked with standard concentrations of these bioactive compounds. Achieving recoveries within this range is crucial for ensuring the reliability and precision of the analytical process when quantifying these essential components in sesame oil. The precision of the whole method was good since the RSDs of the intra-day and inter-day precisions obtained were less than 5%. This indicated that the method is suitable for the precise analysis of phytochemicals in sesame oil.

### 3.3. Analysis of Lignans, Tocopherols, Squalene, and Phytosterol in Sesame Oil Samples

**Figure 2:**
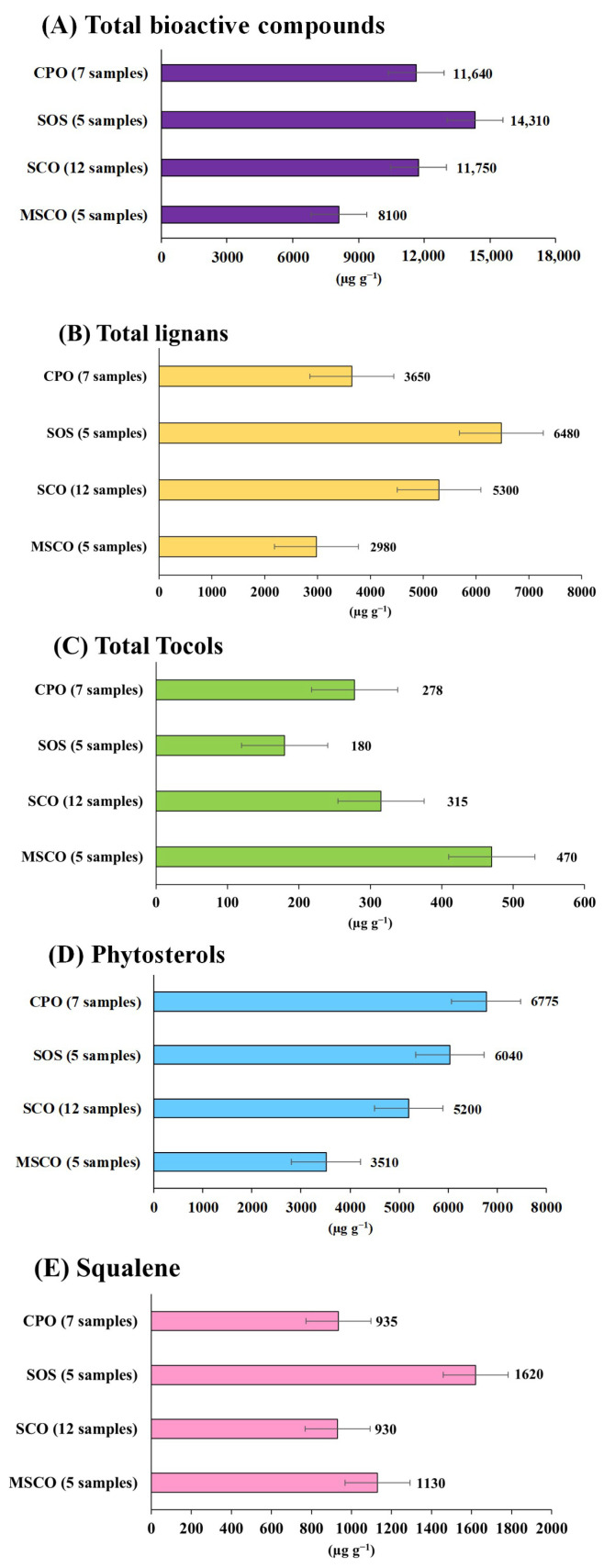
Content of bioactive compounds in sesame oils (μg g^−1^).

The quantification of functional compounds in twenty-eight sesame oil samples was performed and reported in Table 3 and Figure 2. It was found that the contents of the analytes in sesame oil samples were diversified depending on the type and variety of sesame oil, which have been categorized into 4 groups: cold-pressed sesame oil (CPO), sesame oil supplement (SOS), sesame cooking oil (SCO), and sesame cooking oil mixed with other oil (MSCO). Lignans were found in the range of 1775–8965 µg g^−1^, in which the average lignan content was 4644 µg g^−1^. The predominant lignans in sesame oil were sesamin (1210–4725 µg g^−1^) and sesamolin (420–4240 µg g^−1^), while sesamol and asarinin were found in relatively low amounts (non-detectable–121.5 µg g^−1^ for sesamol, and non-detectable–323.8 µg g^−1^ for asarinin). Regarding the category of sesame oil, SOS had the highest lignan content with an average of 6480 µg g^−1^. This would be partly due to its packaging, in which the oil was filled in a yellowish soft gel and packed in an opaque container for protection against photooxidation. Meanwhile, CPO samples had a lower content of lignans (average of 3650 µg g^−1^), which might be due to the clear glass bottle bearing lower protection against environmental light. On the other hand, asarinin and sesamol were observed only in groups of cooking oil (both SCO and MSCO). This was because the refining process of cooking oil involved the addition of acid with thermal processing leading to acidolysis, a process that can partially convert sesamin to asarinin and sesamolin to sesamol [27]. Our results were in accordance with other studies demonstrating that sesamin is the predominant lignan present in sesame oils. Liu et al. reported 633.38 µg g^−1^ of sesamin, with the total lignan content being 908.41 µg g^−1^ [28]. It has been demonstrated that the amount of sesamin is greatly diversified by the variety and cultivation conditions of sesame trees. For instance, a study by Kim et al. and Wang et al. reported a wide range of sesamin (1630–8450 µg g^−1^) [29,30], and sesamolin (200–4300 µg g^−1^) content in sesame seeds [31]. In our study, asarinin was observed only in cooking oil ranging from 18 to 323.8 µg g^−1^, while a study by Huang et al. found asarinin in sesame oil ranging from 12.5 to 664.8 µg g^−1^ [25].

Tocopherol contents in sesame oil samples were 29.7–687.9 µg g^−1^ (average of 314.0 µg g^−1^). γ-Tocopherol was the most abundant form, while β- and δ-tocopherol were almost absent. The MSCO samples appeared to have a greater amount of tocopherols (average of 470 µg g^−1^), while the content in CPO, SOS, and SCO were 278, 180, and 315 µg g^−1^, respectively. Greater tocopherol content in MSCO, including the presence of α, β, and δ-tocopherol, can be attributed in part to the addition of other oils like soybean oil. This is because soybean oil typically contains all four isoforms of tocopherol (α-, β-, γ-, and δ-tocopherols). This implied that sesame oil is not a favorable source of natural vitamin E compared with other vegetable oils. The observed tocopherol content (Table 3) was comparable with other studies. For instance, a study by Huang et al. found the total tocopherol content of sesame oil ranges from 330 to 1010 µg g^−1^ [25]. Williamson et al. reported a range of α-tocopherol around 0.034–0.175 µg g^−1^, δ-tocopherol at 0.44–3.05 µg g^−1^, and γ-Tocopherols at 56.9–99.3 µg g^−1^, respectively [32].

Phytosterol and squalene content was determined in a range of 2640–9500 µg g^−1^ and 245–4030 µg g^−1^, for phytosterol and squalene, respectively. Phytosterol tended to be more abundant in CPO and SOS than SCO and MSCO samples, implying that the preparation process of cooking oil may not be a good extraction process for sesame phytosterol. In contrast, squalene was found to be rather high in the MSCO group, suggesting that the addition of other oils could have increased the squalene content more than that of pure sesame oil. A report by Mohamed et al. showed the highest content (4000–4130 µg g^−1^) of phytosterol in sesame oil, in which β-sitosterol was the most abundant sterol (2317–3052 µg g^−1^) followed by campesterol and stigmasterol [33]. Squalene observed in black sesame oils was 572–1171 µg g^−1^, while white sesame oil was found at 607–2611 µg g^−1^ [18,34]. Furthermore, a comparison of the amounts of functional compounds in sesame oils derived from black sesame seeds and white sesame seeds was observed. Based on our result, there were no obvious differences in the amount of lignans, tocopherols, squalene, and phytosterol between the oils of sesame seeds. The higher popularity of black sesame products than white sesame products could be related to the higher content of minerals such as calcium, potassium, copper, iron, and manganese [35]. For example, the content of calcium and potassium in black sesame seeds was found at 2017.89 and 741.49 mg 100 g^−1^ and 985.54 and 630.66 mg100 g^−1^ in white sesame oil, respectively [36].

Furthermore, the bioactive compounds of roasted and non-roasted sesame seeds in sesame cooking oil (SCO group) were compared. Tocopherol contents in sesame oil from both roasted and non-roasted sesame seeds were quite indifferent (316 and 313 µg g^−1^, respectively). Roasting sesame seeds improved the extraction of lignans, as the lignan content in the oils was higher (5619 µg g^−1^) than that of the oil from non-roasted seeds (4791 µg g^−1^). In addition, by roasting sesame seeds before oil extraction, squalene contents were decreased (887 and 1000 µg g^−1^ for roasted and non-roasted, respectively), and phytosterol contents were increased (5370 and 4835 µg g^−1^ for roasted and non-roasted, respectively).

### 3.4. Statistical Analysis of Principal Component Analysis (PCA)

**Figure 3:**
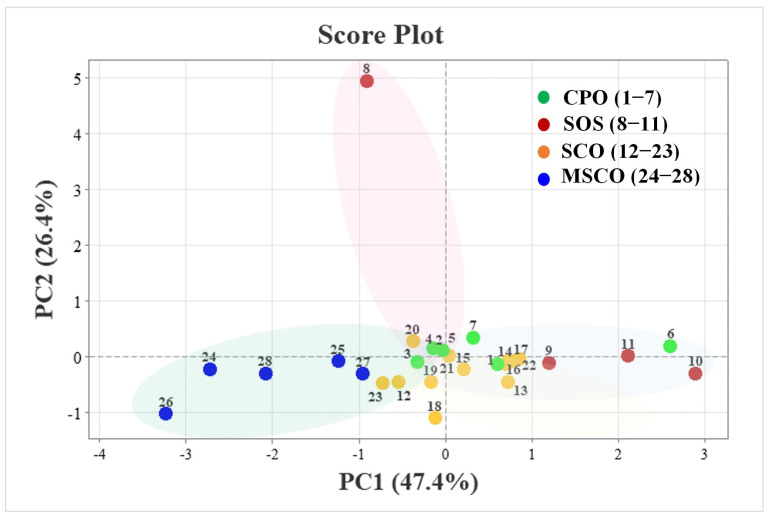
Principal component analysis of the sesame oils for the content of bioactive compounds in the function of PC1 and PC2, score plot (n = 28) for oil samples in 4 groups of cold-pressed sesame oil (CPO), sesame oil supplements (SOS), sesame cooking oil (SCO), and sesame cooking oil mixed with other oils (MSCO). The code for the oils is a number that is counted from the order of the oils written in Supplementary Table S1.

The PCA score plot (Figure 3) showed the sample labels based on the categories of the oil samples. Sesame oil samples from the same categories were found to be similar to each other. For instance, almost all of the MSCO samples tended to be located on the left side of the score plot as the samples in this category had the highest amount of total tocols (470 µg g^−1^), with the sample coded 26 having the highest γ-tocopherol of 529.7 µg g^−1^. On the right side of the score plot, there are clusters of oil that had high contents of lignan and phytosterol, especially in the category of SOS. The sample coded 10 at the far-right side of the sample distribution had a very high content of phytosterol and lignan. However, the CPO coded 6 was scattered outside the main cluster, located in the middle of the score plot. This demonstrated that the sample had the highest phytosterol content among all the samples. Meanwhile, the sample (coded 8) in the SOS category located in the upper part of the score plot exhibited the highest content of squalene. A dense cluster in the middle area of the PCA space were plots of samples of CPO and SCO. Samples in these two categories had a low dispersion because they contained similar amounts of bioactive compounds. The oils from these two groups contained a high composition of sesamin and sesamolin. Among these two groups, coded 18 (belonging to SCO), located at the mid-low area of the PCA space, contained the highest amount of sesamin. The PCA plot suggested a similarity for the analyzed samples as well as highlighting the dissimilarity of the sesame oil profiles. PCA was able to clearly distinguish the different categories of sesame oils in relation to the amount of lignans, tocopherols, phytosterols, and squalene.

## 4. Conclusions

In summary, the simultaneous determination of lignans, tocols, phytosterols, and squalene was established using an HPLC-DAD-FLD system with good linearity, good resolutions, low LOD/ LOQ, and satisfactory recoveries. The method allowed for the analysis of 10 compounds within 22 min. The method validation suggested that the developed method was highly reliable. The method was successfully applied to analyze lignans, tocols, phytosterols, and squalene in twenty-eight commercial sesame oils. The developed HPLC-DAD-FLD method is a powerful tool for the analysis of important bioactive compounds in different types of sesame oil samples (including CPO, SOS, SCO, and MSCO). In addition, the analytical method developed can be applied to other products containing sesame oil for the determination of those phytochemicals.

## Figures and Tables

**Table 1:** Method validation results for determination of lignans, tocopherols, phytosterol, and squalene.

Compounds	Retention Time (min)	Regression Equation	Correlation (R^2^)	LOD ^a^ (µg mL^−1^)	LOQ ^b^ (µg mL^−1^)
Sesamin	10.34	y = 36.705x − 2.918	0.9982	0.02	0.05
Sesamolin	8.94	y =26.738x + 19.573	0.9901	0.10	0.12
Sesamol	11.73	y = 46.016x + 13.691	0.9975	0.02	0.04
Asarinin	9.08	y = 30.685x + 5.4833	0.9947	0.10	0.12
α-Tocopherol	6.31	y = 21.621x − 6.6543	0.9996	0.41	0.43
β-Tocopherol	6.97	y = 41.061x + 3.1872	0.9998	0.35	0.42
γ-Tocopherol	7.52	y = 44.828x + 3.2547	0.9999	0.40	0.42
δ-Tocopherol	8.44	y = 43.128x + 15.908	0.9995	0.21	0.40
Phytosterols	18.17	y = 6.2278x + 60.16	0.9992	10.04	10.34
Squalene	7.38	y = 0.0453x − 5.2587	0.9986	307.07	326.23

LOD ^a^ and LOQ ^b^ are limit of detection (signal-to-noise ratio of 3) and quantification (signal-to-noise ratio of 10), respectively.

**Table 2:** Values of the intra-day, inter-day, and recoveries of the analytes.

Compounds	Concentration (µg mL^−1^)	Intra-Day %RSD (n = 6)	Inter-Day %RSD (n = 3)	Recovery (%)
Sesamin	Con 1	0.18	0.61	107.2
	Con 2	1.09	3.07	100.4
	Con 3	0.78	3.02	104.8
Sesamolin	Con 1	0.14	0.66	99.3
	Con 2	0.35	1.39	100.2
	Con 3	0.27	3.32	97.8
Sesamol	Con 1	0.04	0.22	91.6
	Con 2	0.14	0.39	102.8
	Con 3	0.27	0.14	88.4
Asarinin	Con 1	0.12	0.40	101.1
	Con 2	0.42	1.46	99.7
	Con 3	0.46	2.65	93.5
α-Tocopherol	Con 1	0.27	0.03	92.5
	Con 2	0.16	0.45	88.9
	Con 3	0.33	0.26	96.7
β-Tocopherol	Con 1	0.28	0.10	102.0
	Con 2	0.22	0.25	109.4
	Con 3	0.30	0.36	113.5
γ-Tocopherol	Con 1	0.08	0.35	100.0
	Con 2	0.12	0.34	98.5
	Con 3	0.19	0.33	107.6
δ-Tocopherol	Con 1	0.07	0.11	102.6
	Con 2	0.07	0.13	90.6
	Con 3	0.16	0.34	91.4
Squalene	Con 1	0.04	0.30	99.7
	Con 2	0.10	0.28	100.9
	Con 3	0.22	0.46	104.7
Phytosterol	Con 1	0.01	0.10	91.0
	Con 2	0.04	0.13	92.5
	Con 3	0.03	0.25	94.6

**Table 3:** Content of lignans, tocopherols, phytosterol, and squalene in various commercialized sesame oil samples (μg g^−1^).

Sample	Tocopherol				Lignan				Squalene	Phytosterols
	α-T ^a^	β-T	γ-T	δ-T	Sesamolin	Asarinin	Sesamin	Sesamol		
CPO-1	ND ^b^	ND	293.8	ND	2240	ND	2315	ND	875	6640
CPO-2	ND	ND	286.6	ND	1275	ND	1260	ND	935	6385
CPO-3	ND	ND	377.5	ND	1540	ND	1480	ND	1005	6690
CPO-4	ND	ND	298.9	ND	1300	ND	1280	ND	960	6530
CPO-5	ND	ND	324.8	ND	1645	ND	1620	ND	1030	6610
CPO-6	ND	ND	194.2	ND	2800	ND	2990	ND	845	9500
CPO-7	ND	ND	169.4	ND	1820	ND	1940	ND	910	5065
SOS-1	ND	ND	162.0	1.3	1630	ND	1630	ND	4030	4885
SOS-2	ND	ND	249.4	ND	3330	ND	3840	ND	995	5490
SOS-3	ND	ND	29.7	ND	3000	ND	3540	ND	245	7055
SOS-4	ND	ND	266.9	ND	4240	ND	4725	ND	1195	6725
SCO-1	38.3	5.4	298.8	ND	2130	92.8	2495	7.6	875	4120
SCO-2	ND	ND	391.6	ND	2615	122.8	3215	25.1	965	7020
SCO-3	ND	ND	329.2	ND	2365	115.2	2690	9.5	990	6900
SCO-4	ND	ND	250.9	ND	2175	168.9	2740	7.9	815	4560
SCO-5	ND	ND	254.1	ND	2500	41.8	2900	5.5	905	5850
SCO-6	ND	ND	275.3	ND	2850	132.9	3360	121.5	1040	5210
SCO-7	147.8	ND	226.4	ND	1730	323.8	4360	30.5	620	3930
SCO-8	ND	ND	400.7	ND	2300	157.8	2740	30.0	990	5495
SCO-9	ND	ND	307.5	ND	1500	18.0	2980	42.8	1260	4610
SCO-10	ND	ND	270.0	ND	2140	126.1	2440	20.4	990	4835
SCO-11	ND	ND	283.0	ND	2860	60.3	3180	105.3	1050	5895
SCO-12	80.0	18.9	206.7	ND	1490	12.7	2115	ND	705	4000
MSCO-1	100.3	12.6	343.9	ND	420	145.2	1210	ND	1160	2640
MSCO-2	53.2	14.1	311.9	ND	1390	104.5	1765	32.0	1115	4195
MSCO-3	139.4	18.8	529.7	ND	685	213.1	1555	1.7	1175	3640
MSCO-4	57.2	21.8	308.7	ND	1800	150.4	2525	7.8	1070	3995
MSCO-5	67.8	14.7	357.9	ND	1215	183.5	1515	30.6	1130	3090

T ^a^ = tocopherol and ND ^b^ = non-detectable.

## Data Availability

The original contributions presented in the study are included in the article, further inquiries can be directed to the corresponding author.

## Supplementary Materials

The following supporting information can be downloaded at https://www.mdpi.com/article/10.3390/foods13091368/s1. The supplementary information available includes a table and chromatograms derived from the four HPLC conditions. Table S1: Detail of sesame oil samples used in this study. Figure S1: Chromatograms of some mobile phase ratios showed effective in the separation of the targeted compounds from the four conditions.

## Abbreviations

CPO	Cold-pressed sesame oil
DAD	Diode array detector
FLD	Fluorescence detector
HPLC	High-performance liquid chromatography
LOD	Limit of detection
LOQ	Limit of quantification
MSCO	Sesame cooking oil mixed with other oil
NP	Normal-phase
PCA	Principal component analysis
RP	Reverse-phase
RSDs	Relative standard deviations
SCO	Sesame cooking oil
SOS	Sesame oil supplement
