# Supplementary material for: Development of a Simultaneous Normal-Phase HPLC Analysis of Lignans, Tocopherols, Phytosterols, and Squalene in Sesame Oil Samples
Source: Foods. 2024 Apr 28;13(9):1368. doi: 10.3390/foods13091368 (PMC11083186; doi:10.3390/foods13091368)
Supplement: Supplementary file 1 [file foods-13-01368-s001.zip › foods-2970182-supplementary.pdf]

Foods

Article

Supplementary materials

Development of a Simultaneous Normal-Phase HPLC Analysis of Lignans, Tocopherols, Phytosterols and Squalene in Sesame Oil Samples

Jitkunya Yuenyong[1,2], Chonlada Bennett[1,3], Sudarat Jiamyangyuen[4], Sugunya Mahatheeranont[1,3,5,6], and Phumon Sookwong[1,3,6*]

Supplementary Table 1. Detail of sesame oil samples used in this study.

Sample SO	Description	Source
**CPO-1 (1)**	Cold-pressed white sesame oil (100%)	Thailand
**CPO-2 (2)**	Cold-pressed black sesame oil (100%)	Thailand
**CPO-3 (3)**	Cold-pressed black sesame oil (100%)	Thailand
**CPO-4 (4)**	Cold-pressed black sesame oil (100%)	Thailand
**CPO-5 (5)**	Cold-pressed black sesame oil (100%)	Thailand
**CPO-6 (6)**	Cold-pressed black sesame oil (100%)	Thailand
**CPO-7 (7)**	Cold-pressed black sesame oil (100%)	Thailand
**SOS-1 (8)**	Cold-pressed black sesame oil (100%) in soft gel	Thailand
**SOS-2 (9)**	Cold-pressed black sesame oil (100%) in soft gel	Thailand
**SOS-3 (10)**	Cold-pressed black sesame oil (100%) in soft gel	Thailand
**SOS-4 (11)**	Cold-pressed black sesame oil (100%) in soft gel	Thailand
**SCO-1 (12)**	Sesame oil from roasted white sesame seeds	China
**SCO-2 (13)**	Sesame oil from roasted white sesame seeds	Korea
**SCO-3 (14)**	Sesame oil from roasted white sesame seeds	Korea
**SCO-4 (15)**	Sesame oil from roasted white sesame seeds	Japan
**SCO-5 (16)**	Sesame oil from roasted black sesame seeds	China
**SCO-6 (17)**	Sesame oil from roasted black sesame seeds	China
**SCO-7 (18)**	Sesame oil from roasted black sesame seeds	China
**SCO-8 (19)**	Sesame oil from roasted white sesame seeds (95%) +black sesame seeds (5%)	China
**SCO-9 (20)**	White sesame oil (100%)	Thailand
**SCO-10 (21)**	White sesame oil (100%)	Thailand
**SCO-11 (22)**	Black sesame oil (100%)	India
**SCO-12 (23)**	Black sesame oil (100%)	Singapore
**MSCO-1 (24)**	White sesame oil (50%) +soybean oil (50%)	Singapore
**MSCO-2 (25)**	Black sesame oil (70%) +soybean oil (30%)	Thailand
**MSCO-3 (26)**	Black sesame oil (70%) +soybean oil (30%)	Thailand
**MSCO-4 (27)**	Black sesame oil (80%) +soybean oil (20%)	Thailand
**MSCO-5 (28)**	Sesame oil from roasted black sesame seeds (80%) +soybean oil (20%)	China

Supplementary Figure 1. Chromatograms of some mobile phase ratios showed effective in the separation of the targeted compounds from the four conditions.

A

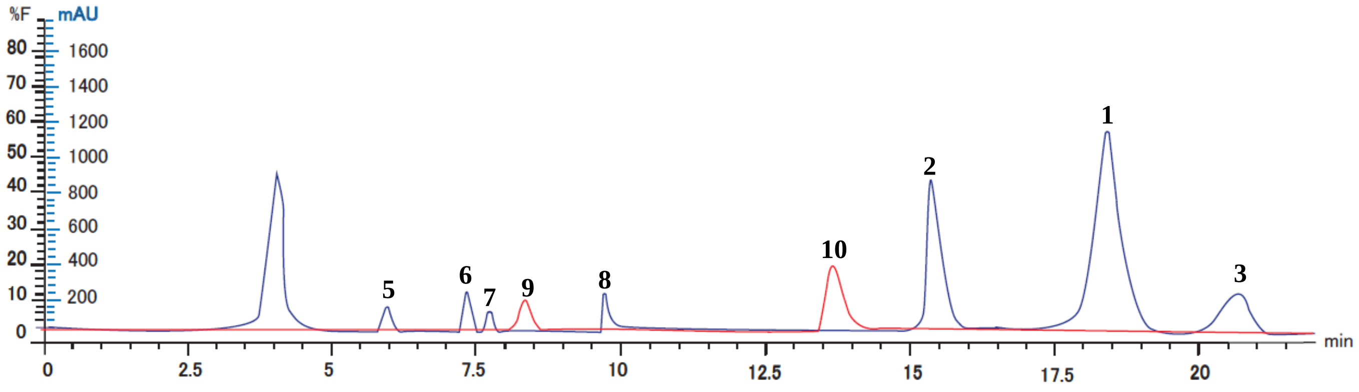

Condition A, a mixture of n-hexane/tetrahydrofuran/acetonitrile/2-propanol (93:6:0.5:0.5, v/v/v/v)

using a flow rate of 0.8 mL min^{-1}

B

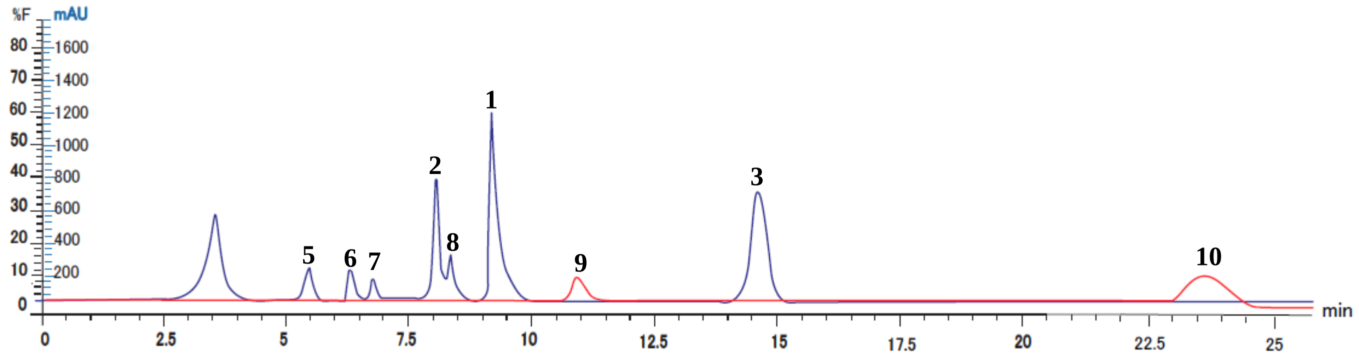

Condition B, a mixture of n-hexane/tetrahydrofuran/acetonitrile/2-propanol (92:6:1:1, v/v/v/v)

using a flow rate of 0.5 mL min^{-1}.

C

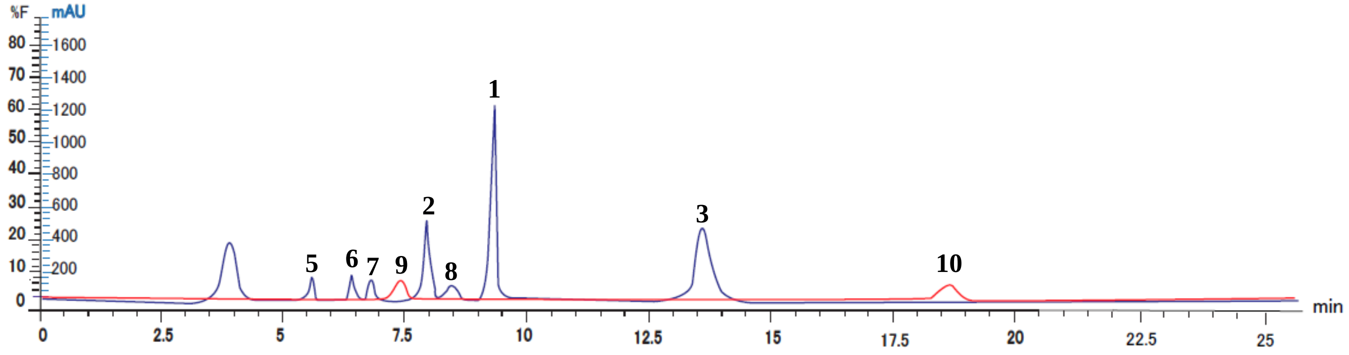

Condition C, a mixture of n-hexane/tetrahydrofuran/acetonitrile (93:6:1, v/v/v)

using a flow rate of 1.2 of mL min^{-1}

D

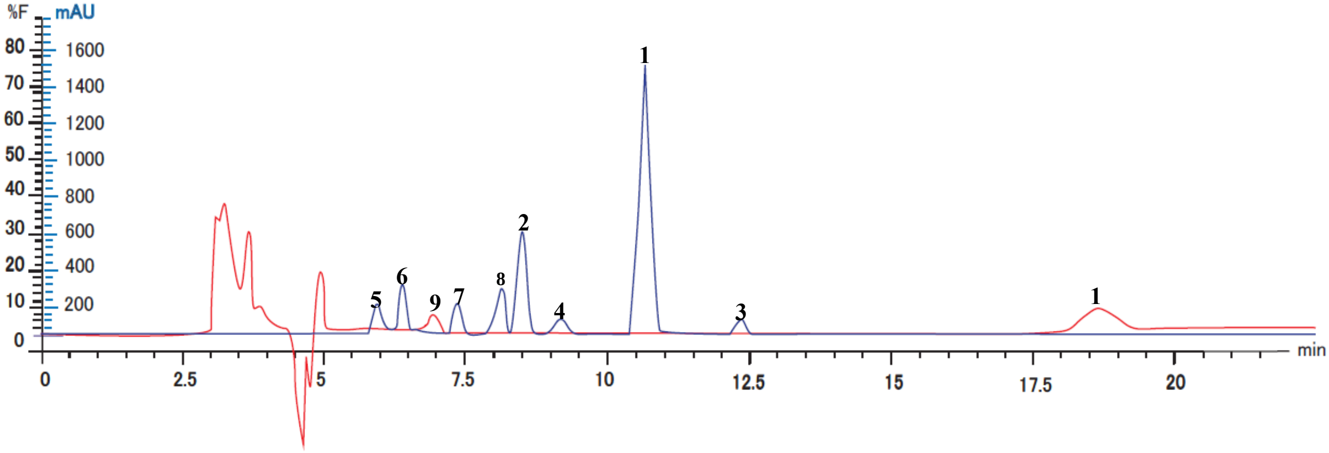

Condition D, a mixture of n-hexane/tetrahydrofuran/2-propanol (93:6:1, v/v/v)

using a flow rate of 0.8 of mL min^{-1}

Peak identification: 1 = Sesamin, 2 = Sesamolin, 3 = Sesamol, 4 = Asarinin, 5 = α-Tocopherol, 6 = β-Tocopherol, 7 = γ-Tocopherol, 8 = δ-Tocopherol, 9 = Squalene, and 10 = Phytosterol.
